# Measuring Gratitude: A Comparative Validation of the Dutch Gratitude Questionnaire (GQ6) and Short Gratitude, Resentment, and Appreciation Test (SGRAT)

**DOI:** 10.5334/pb.bd

**Published:** 2015-05-15

**Authors:** Lilian Jans-Beken, Johan Lataster, Roeslan Leontjevas, Nele Jacobs

**Affiliations:** 1 Faculty of Psychology and Educational Sciences, Open University; Heerlen, The Netherlands; 2 Department of Psychiatry and Psychology, School for Mental Health and Neuroscience, Maastricht University Medical Centre; Maastricht, The Netherlands

**Keywords:** gratitude, life satisfaction, positive affect, negative affect, validation, Dutch

## Abstract

The aim of this article was to validate and compare the Dutch translations of the Gratitude Questionnaire (GQ6) and the Short Gratitude, Resentment, and Appreciation Test (SGRAT) in an adult general population sample. In an online survey, 706 respondents (M_*age*_ = 44, SD_*age*_ = 14) completed Dutch versions of the GQ6, the SGRAT, the Satisfaction With Life Scale (SWLS) and the Positive Affect and Negative Affect Schedule (PANAS). At six week follow-up, 440 (62%) of them (M_*age*_ = 46, SD_*age*_ = 14) again completed the GQ6-NL and SGRAT-NL. Parallel analyses, exploratory factor analyses and confirmatory factor analyses revealed and confirmed one factor for the GQ6-NL, and three factors for the SGRAT-NL. Internal consistency indices of the GQ6-NL and of the SGRAT-NL were satisfactory. Both questionnaires demonstrated good test-retest reliability. Regression analyses showed, for the total scores on both gratitude questionnaires, positive associations with the SWLS and the Positive Affect Scale, and negative associations with the Negative Affect Scale. The results support the validity of the Dutch GQ6 and SGRAT. These questionnaires can be used to conduct further research of the grateful disposition in Dutch speaking individuals and groups.

This article describes the validation study of the Dutch translations of the Gratitude Questionnaire (GQ6; McCullough, Emmons, & Tsang, 2002) and the Short Gratitude, Resentment, and Appreciation Test (SGRAT; Thomas & Watkins, 2003). These questionnaires were developed to measure the grateful disposition which is defined as a ‘generalized tendency to recognize and respond with grateful emotion to the roles of other people’s benevolence in the positive experiences and outcomes that one obtains’ (McCullough et al., 2002, p. 112).

McCullough et al. (2002) proposed a theoretical framework wherein four facets of gratitude are distinguished: intensity, frequency, span, and density. An individual with a strong grateful disposition is thought to experience gratitude more intensely and more frequently than someone with a weaker grateful disposition. Span refers to the number of life events for which a person feels grateful at a given time, and density refers to the number of persons one is grateful to. McCullough et al. (2002) developed the GQ6 based on these four facets.

Another theoretical framework was proposed by Watkins, Woodward, Stone, and Kolts (2003), identifying three distinct characteristics within a grateful individual. The first characteristic is a lack of a sense of deprivation. The second characteristic is the tendency to appreciate simple pleasures, and the third characteristic is the tendency to appreciate the contributions of others to one’s own well-being and to express this gratitude. Watkins et al. (2003) developed the SGRAT based on these three characteristics.

Recent empirical studies have shown positive associations of the grateful disposition with subjective well-being (Emmons & McCullough, 2003; McCullough et al., 2002; Thomas & Watkins, 2003; Watkins et al., 2003; Wood, Froh, & Geraghty, 2010), happiness (Watkins et al., 2003), spiritual transcendence (Diessner & Lewis, 2007), religiousness and spirituality (McCullough et al., 2002), optimism (Chen, Chen, Kee, & Tsai, 2009), and positive affect (Emmons & McCullough, 2003; McCullough et al., 2002; Thomas & Watkins, 2003; Watkins et al., 2003). Negative associations have been found between the grateful disposition and depression (Thomas & Watkins, 2003; Watkins et al., 2003), negative affect (Thomas & Watkins, 2003), and aggression (Watkins et al., 2003). These correlates have in turn been causally linked to cardiovascular disease (Krantz, Contrada, Hill, & Friedler, 1988; Suinn, 2001), hypertension (Shapiro & Goldstein, 1982), and immune system dysfunction (Cohen, Tyrrell, & Smith, 1993; Graham, Christian, & Kiecolt-Glaser, 2006). The results of the aforementioned empirical studies show the importance of dispositional gratitude as a possible protective factor in health care and thereby the importance of measures to assess the grateful disposition.

There are several reasons for validating Dutch translations of the gratitude questionnaires. First, although large numbers of people in the Netherlands and Belgium can speak and understand English, English reading comprehension is strongly associated with socioeconomic status (EF - EPI, 2014). Second, the use of a translated questionnaire prevents responses being affected by cultural accommodation (Harzing, 2005), misinterpretation, and reduces the cognitive and emotional bias that exists when answering questions in another language than one’s mother tongue (Keysar, Hayakawa, & An, 2012). Third, Dutch is the official language in six countries of the world, representing a total population of more than twenty eight million people. Taken together, a questionnaire in Dutch is invaluable for studying gratitude in Dutch-speaking countries. These brief questionnaires were selected for validation because previous research showed them to be reliable and valid measures of the grateful disposition in English-speaking populations (McCullough et al., 2002; Watkins et al., 2003). Particularly, a validation study of two different scales can help the reader to choose the most appropriate scale. The current comparative validation may be valuable for the international reader because the scales are based on different theoretical frameworks and the scales’ comparison adds to the discussion on the grateful disposition as a psychological construct. For the translated scales we aimed to assess their factorial structure, their internal consistency, test-retest reliability, and the convergent, divergent, and concurrent validity.

## Method

### Respondents

We recruited participants mainly through social media, e-mails, personal contacts, and door-to-door flyers with the intention to collect a sample as heterogeneous as possible in terms of gender, age, education, employment status, and religious affiliation. Inclusion criteria were: (a) Dutch speaking, and (b) eighteen years or older. Participants enrolled voluntarily and were rewarded for participation with a raffle for gift cards. In the informed consent, ethical and privacy issues were covered. Confidentiality as well as anonymity were ensured. The convenience sample consisted of 706 Dutch speaking adults at baseline (*M_age_* = 44, *SD_age_* = 14, *Range* = 18 - 80). At follow-up, 440 participants (62%) of the initial sample completed the survey (*M_age_* = 46, *SD_age_* = 14, *Range* = 18 - 80). These subjects (hereafter: completers) were significantly higher educated and older, and reported less negative affect compared to subjects who completed only the baseline survey (hereafter: dropouts; Table 1).

**Table 1:** Sample characteristics at baseline including drop-outs and completers (N = 706).

	Whole sample	Drop-outs	Completers	Δ Drop-out - Completers

*n*	706	266	440	
Low education *N*(%)	173(25)	85(31)	88(20)	χ^2^ (1, *N* = 706) = 10.353^**^
High education *N*(%)	533(75)	181(69)	352(80)	
Full time employed *N*(%)	201(28)	88(33)	114(26)	χ^2^ (2, *N* = 706) = 4.382
Part time employed *N*(%)	241(34)	85(32)	154(35)	
Not employed *N*(%)	264(38)	93(35)	172(39)	
No belief *N*(%)	327(47)	122(46)	202(46)	χ^2^ (2, *N* = 706) = 0.025
Religious *N*(%)	234(33)	91(34)	145(33)	
Spiritual *N*(%)	145(20)	53(20)	93(21)	
Age *M(SD)*	43.90(14.10)	40.07 (13.67)	46.22 (13.86)	*t (704)* = -5.74^**^
GQ6-NL *M(SD)*	32.51(5.14)	32.12 (5.14)	32.75 (5.14)	*t (704)* = -1.584
SGRAT-NL *M(SD)*	110.95(15.54)	109.71 (15.49)	111.70 (15.54)	*t (704)* = -1.648
SWLS *M(SD)*	24.58(6.41)	24.03 (6.46)	24.92 (6.36)	*t (704)* = -1.780
PA-scale *M(SD)*	35.40(7.05)	35.33 (7.06)	35.44 (7.05)	*t (704)* = -0.201
NA-scale *M(SD)*	19.21(7.72)	20.15 (7.74)	18.64 (7.66)	*t (704)* = 2.524*

*Note*. ^**^ p < 0.001, ^*^ p < 0.05. Dropouts are participants who completed only the baseline survey. Completers are participants who completed both the baseline and six-week follow-up survey.

### Measures

**Gratitude.** The grateful disposition was measured with Dutch translations of the GQ6 (McCullough et al., 2002), and the SGRAT (Thomas and Watkins, 2003).

***GQ6-NL.*** The GQ6 consists of six propositions representing one single factor with acceptable internal consistency (Cronbach’s α = 0.82) (McCullough et al., 2002). Respondents indicate their response on a 7-point Likert scale, ranging from strongly disagree (1) to strongly agree (7). Two negatively formulated items are reverse coded and item scores are summed to a total score, ranging from 6 to 42, with high scores indicating a higher level of a grateful disposition.

***SGRAT-NL.*** The GRAT was initially developed by Watkins et al. (2003). They conducted four studies to develop and validate this scale consisting of 44 items allocated to three subscales. Thomas and Watkins (2003) revised the GRAT and developed a short form. The remaining 16 items of the SGRAT displayed a Cronbach’s α = 0.92 for the total score. This short version appeared to be as reliable and valid as the initial GRAT. Diessner and Lewis (2007) confirmed the original three-factor structure with factors (a) Lack of a Sense of Deprivation (LOSD), (b) Simple Appreciation (SA), and (c) Appreciation for Others (AO). Respondents indicate their response on a 9-point Likert scale, ranging from strongly disagree (1) to strongly agree (9). Five negatively formulated items are reverse coded. The total score ranges from 16 to 144, and high scores indicate a higher level of the grateful disposition.

**Subjective well-being.** We used the definition of Myers and Diener (1995) for subjective well-being, comprising frequent positive affect, infrequent negative affect, and a sense of life satisfaction.

***Life Satisfaction.*** Life satisfaction is an evaluation of the quality of life according to criteria chosen by the individual (Shin & Johnson, 1978), which was measured with the validated Dutch version of the Satisfaction With Life Scale (SWLS; Arrindell, 1991; Diener, Emmons, & Griffin, 1985). The questionnaire consists of five propositions on which the respondents indicate their response using a 7-point Likert scale, ranging from strongly disagree (1) to strongly agree (7). All item scores are summed to a total score, ranging from 5 to 35, with high scores indicating a higher level of life satisfaction. The SWLS is found to be a reliable measure with reported Cronbach’s α values in the range of 0.85 to 0.87 (Arrindell, 1991; Van Beuningen, 2012).

***Positive and negative affect.*** Affect was measured with the validated Dutch Positive Affect and Negative Affect Schedule (PANAS; Peeters, Ponds, & Vermeeren, 1996). The schedule measures two dimensions: positive affect and negative affect. The questionnaire consists of twenty descriptor terms: ten items measuring positive affect, and ten items measuring negative affect. Respondents are asked to rate the extent to which they have experienced each mood state during the past week on a 5-point Likert scale, ranging from very slightly or not at all (1) to extremely (5). Scores on each dimension are summed to a total score, ranging from 10 to 50 for each dimension, with high scores indicating a higher level of positive or negative affect. Dutch translations of the negative affect scale (NA-scale) and positive affect scale (PA-scale) showed internal consistencies of α = 0.83 and α = 0.79, respectively (Peeters et al., 1996).

### Procedure

Both the GQ6 and the SGRAT were translated into Dutch by a translator who was raised bilingual. A second bilingual translator translated the Dutch items back into English. Dutch and English items were evaluated by both translators and the researcher to ensure equivalence in meaning and comparability of the items. The items of the translated SGRAT and GQ6 are listed in Table 2 and Table 3, respectively. Study participants filled in an online survey at baseline (GQ6, SGRAT, SWLS, and the PANAS) and at six-weeks follow-up (GQ6 and SGRAT).

**Table 2:** Short Gratitude, Resentment and Appreciation Test, Dutch translation (S-GRAT-NL).

Item	

1	Zonder de hulp van veel mensen had ik niet kunnen komen waar ik nu ben in mijn leven.
2	Het leven is goed voor me geweest.
3	Het lijkt alsof er nooit genoeg is waardoor ik mijn deel nooit krijg.
4	Ik ben vaak overweldigd door de schoonheid van de natuur.
5	Ik vind dat het niet alleen belangrijk is om trots te zijn op mijn prestaties maar ook te herinneren welke rol anderen hebben gespeeld bij het tot stand komen van de prestaties.
6	Ik denk niet dat ik alle goede dingen heb gekregen die ik verdien in het leven.
7	Elke herfst geniet ik echt van de bladeren die van kleur veranderen.
8	Ondanks dat ik de controle heb over mijn leven, denk ik toch veel aan de mensen die me hebben aangemoedigd en geholpen.
9	Het is belangrijk om af en toe stil te staan bij de mooie dingen in het leven.
10	Er zijn meer slechte dingen gebeurd in mijn leven dan dat ik verdien.
11	Door alles wat ik heb meegemaakt in mijn leven, vind ik dat de wereld me iets verschuldigd is.
12	Het is belangrijk om je zegeningen te tellen.
13	Het is belangrijk om te genieten van de simpele dingen in het leven.
14	Ik ben zeer dankbaar voor alle dingen die andere mensen voor me hebben gedaan in mijn leven.
15	Om de een of andere reden krijg ik niet de voordelen die anderen wel krijgen.
16	Het is belangrijk om iedere dag dat je leeft te waarderen.

Note. Items 3, 6, 10, 11, and 15 should be reverse coded. Items 2, 3, 6, 10, 11, and 15 constitute the Lack of a Sense of Deprivation (LOSD) factor. Items 4, 7, 9, 12, 13, and 16 constitute the Simple Appreciation (SA) factor. Items 1, 5, 8, and 14 constitute the Appreciation for Others (AO) factor. Answers are scored on a 9-point Likert scale: (1) *Sterk mee oneens*, (3) *Enigszins mee oneens*, (5) *Neutraal*, (7) *Enigszins mee eens*, (9) *Sterk mee eens.*

**Table 3:** Gratitude Questionnaire-6, Dutch translation (GQ-6-NL).

Item	

1	Ik heb veel dingen in het leven om dankbaar voor te zijn.
2	Als ik een lijst zou maken van alle dingen waar ik dankbaar voor ben, wordt dat een hele lange lijst.
3	Als ik naar de wereld kijk, zijn er niet veel dingen om dankbaar voor te zijn.
4	Ik ben veel verschillende mensen dankbaar.
5	Naarmate ik ouder word, kan ik mensen, gebeurtenissen en situaties die deel van mijn leven zijn, meer waarderen.
6	Het duurt soms lang voor ik dankbaar kan zijn voor iets of iemand.

*Note.* Items 3 and 6 should be reverse coded. Answers are scored on a 7-point Likert scale: (1) *Sterk mee oneens*, (2) *Mee oneens*, (3) *Enigszins mee oneens*, (4) *Neutraal*, (5) *Enigszins mee eens*, (6) *Mee eens*, (7) *Sterk mee eens.*

### Analyses

Differences in demographic variables, as well as in the main variables of gratitude and subjective well-being between completers and dropouts, were examined using chi-square tests for categorical variables and independent t-tests for continuous variables. Parallel analysis with Monte Carlo simulations was conducted on the items at baseline (T0) of the GQ6-NL and SGRAT-NL in order to determine the number of factors to retain in Exploratory Factor Analysis (Horn, 1965). The simulation was executed with 1000 parallel datasets based on permutations of the original raw data set, with the criterion set at the 95^th^ percentile. The eigenvalue of the raw data needed to exceed the eigenvalue of the 95^th^ percentile to be defined as a factor (O’Connor, 2000). Exploratory Factor Analyses (EFA) using maximum-likelihood were applied on the items of the GQ6-NL and SGRAT-NL at baseline (T0). To assess the sampling adequacy, a Kaiser-Meyer-Olkin (KMO) measure was conducted. A KMO is considered good when the outcome is between 0.7 - 0.8, and excellent when between 0.8 - 0.9 (Hutcheson, & Sofroniou, 1999). Anti-image correlations of > 0.5 were regarded acceptable (Field, 2013). Factor loadings were examined, and rotation of factors with direct oblimin was applied when more than one factor was found. Confirmatory factor analyses (CFA) using maximum-likelihood estimation were applied on respectively the items of the GQ6-NL and SGRAT-NL to confirm the factor structures of the questionnaires at six weeks follow-up (T1). To assess goodness of fit, the chi-square (χ^2^), comparative fit index (CFI) and standardized root-mean-square residual (SRMR) statistics were examined. CFI values above 0.95 and SRMR values below 0.05 are typically considered to indicate that a model is adequately parameterized although values as high as 0.90 and as low as 0.10 are acceptable (Hu & Bentler, 1999). Internal consistency was determined by McDonald’s omega (ω_h_), accounting for the proportion of variance a potential latent variable explains on a general factor (Zinbarg, Revelle, Yovel, & Li, 2005). McDonald’s omega values between 0.70 and 0.80 were considered acceptable, and between 0.80 and 0.90 as good (Terwee et al., 2007). The test-retest reliability was evaluated using the intraclass correlation coefficient (ICC) with a two-way random effects model with absolute agreement (Shrout & Fleiss, 1979). An ICC over 0.70 can be considered good in a sample with at least 50 cases (Terwee et al., 2007). Regression analyses were performed to test for convergent and divergent validity. For convergent validity, (1) total scores of gratitude scales, and (2) SGRAT-NL subscales were used as predictors of the SWLS and PA scores. To assess divergent validity, regression analyses were conducted for the NA scale using (1) gratitude scales’ total scores, and (2) SGRAT-NL subscales as predictors. Regarding the subscales, we controlled for the variance inflation factor (VIF < 10) , and a tolerance of more than 0.1 to preclude multicollinearity (Fields, 2013). For convergent validity it was expected that the beta for the associations between gratitude (sub)scores measure with the GQ6 and SGRAT-NL and SWLS and PA would be positive and between 0.40 and 0.59 (Evans, 1996); for divergent validity a negative or no association was expected between gratitude (sub)scores measured with the GQ6 and SGRAT-NL and NA. To test for concurrent validity between gratitude scales, Pearson’s correlation coefficient was calculated at T0 and T1. It was expected that the Pearson’s *r* would be positive and 0.70 or greater (Terwee et al., 2007). All results were interpreted against a significance threshold of 5%, and 95% confidence intervals were calculated. Analyses were conducted using SPSS 20.0 except for the CFA and McDonalds omega, which were conducted using Lavaan 0.5–16 (Rosseel, 2012) in R 3.0.3.

## Results

### GQ6-NL

Parallel analysis showed one factor for the GQ6-NL (Table 4). The KMO of 0.74 verified the sampling adequacy for the EFA at T0. Anti-image correlation values for individual items were all ≥ 0.70, which is well above the acceptable limit of 0.50. All but item six loaded satisfactory on the single factor (Table 5). Rotation was not conducted because of the one-factor scale of the GQ6-NL. Our CFA confirmed the one-factor structure of the GQ6-NL at T1 with a good fit with the sample, χ^2^ (9, *N* = 444) = 65.752, *p* < 0.001, CFI = 0.92, SRMR = 0.06. Internal consistency was acceptable, ω_h_ = 0.75. Item six was retained in the factor because at least three items within the factor showed high loadings (Pasta & Suhr, 2004), all items had a good anti-image correlation, internal consistency of the factor did not improve with at least 0.05 when item 6 was removed (ω_h_ = 0.77), and CFA confirmed the one-factor structure. The test-retest reliability for the GQ6-NL was good (Table 6). Results of the regression analysis showed that the total score of the GQ6-NL was moderately positively associated with life satisfaction and positive affect, and moderately to weakly negatively associated with negative affect (Table 7).

**Table 4:** Parallel analyses from the items of the GQ6-NL and SGRAT-NL (N = 706).

Measures and factors	Raw data	95^th^ percentile	Proportion variance explained (%)

GQ6-NL
– Factor 1	2.668317	1.037477	35.68
– Factor 2	1.176871	1.176871	
SGRAT-NL
– Factor 1	4.350429	1.315935	23.74
– Factor 2	2.853171	1.246335	14.93
– Factor 3	1.861935	1.199335	8.43
– Factor 4	0.978189	1.157936	

*Note*. Parallel analyses with Monte Carlo simulations determines the number of factors to retain in Exploratory Factor Analysis (Ledesma & Valero-Mora, 2007). The simulation was executed with 1000 parallel datasets based on permutations of the original raw data set, with the criterion set at the 95^th^ percentile. The eigenvalue of the raw data needs to exceed the eigenvalue of the 95^th^ percentile to be defined as a factor (O’Connor, 2000).

**Table 5:** Factor matrix with loadings of GQ6-NL items (N = 706).

Item	Factor 1

Item 2	0.889
Item 1	0.795
Item 4	0.488
Item 5	0.436
Item 3	0.412
Item 6	0.347

*Note*. Extraction Method: Maximum Likelihood.

**Table 6:** Test-retest reliability after a six week interval of the GQ6-NL and SGRAT-NL.

Measures	ICC(2,2)	CI

GQ6-NL	0.85^**^	0.82 – 0.88
SGRAT-NL	0.91^**^	0.89 – 0.92
LOSD	0.89^**^	0.87 – 0.91
SA	0.89^**^	0.87 – 0.91
AO	0.89^**^	0.86 – 0.91

*Note*. ^**^ *p* < 0.001, ICC = intraclass correlation coefficient, CI = confidence interval, LOSD = Lack of a sense of deprivation, SA = Simple appreciation, AO = Appreciation of others.

**Table 7:** Multiple regression coefficients of the independent variables GQ6-NL, SGRAT-NL, and subscales, and the dependent variables SWLS, PA-scale, and NA-scale.

	SWLS				PA				NA			

	B-coefficient (Standard Error)	CI	Beta	R^2^	B-coefficient (Standard Error)	CI	Beta	R^2^	B-coefficient (Standard Error)	CI	Beta	R^2^

Model 1				0.19**				0.23**				0.07**
GQ6-NL	0.542 (0.042)	0.459 - 0.625	0.44**		0.651 (0.045)	0.562 - 0.741	0.48**		-0.400 (0.055)	-0.507 --0.293	-0.27**	
Model 2				0.26**				0.14**				0.08**
SGRAT-NL	0.212 (0.013)	0.186 - 0.238	0.51**		0.171 (0.016)	0.141 - 0.203	0.38**		-0.143 (0.018)	-0.178 -0.107	-0.29**	
Model 3				0.31**				0.15**				0.14**
SGRAT-NL subscales											
LOSD	0.316 (0.021)	0.275 - 0.358	0.48**		0.141 (0.026)	0.090 - 0.192	0.20**		-0.257 (0.028)	-0.313 -0.201	-0.33**	
SA	0.169 (0.034)	0.103 - 0.235	0.17**		0.247 (0.041)	0.166 - 0.328	0.22**		-0.177 (0.045)	-0.266 -0.088	-0.15**	
AO	0.054 (0.034)	-0.013 - 0.120	0.05		0.149 (0.041)	0.068 - 0.230	0.13**		0.122 (0.046)	0.033 - 0.212	0.10 *	

*Note*. N = 706, ^*^ *p* < 0.05, ^**^ *p* < 0.001. CI = 95% confidence Interval, R^2^ = percentage variance explained. SWLS = Satisfaction With Life Scale; SGRAT-NL = Short Gratitude, Resentment, and Appreciation Test; PA = positive affect scale; NA = negative affect scale; LOSD = lack of a sense of deprivation; SA = simple appreciation; AO = appreciation of others.

### SGRAT-NL

Parallel analysis showed three factors for the SGRAT-NL (Table 4). The KMO of 0.84 verified the sampling adequacy for the EFA at T0. Anti-image correlation values for individual items were ≥ 0.77, which is well above the acceptable limit of 0.50. The rotated component matrix showed that all items of a specific subscale loaded on the same factor (Table 8) corresponding with the subscales of the original SGRAT. CFA on T1 confirmed the three-factor structure of the SGRAT-NL with acceptable fit, χ^2^ (101, *N* = 444) = 481.800, *p* < 0.001, CFI = 0.88, SRMR = 0.07. The internal consistency of the subscales of the SGRAT-NL was good (LOSD ω_h_ = 0.86, SA ω_h_ = 0.79, AO ω_h_ = 0.82). The total SGRAT-NL also showed good internal consistency, ω_h_ = 0.88. Test-retest reliability showed good results for the total score and for all subscales (Table 6). VIF and tolerance scores indicated no concern about multicollinearity. The total score of the SGRAT-NL was moderately positively associated with life satisfaction and positive affect, and moderately to weakly negatively associated with negative affect. When controlled for the separate contribution of all other SGRAT-NL subscale measures, scores on the LOSD subscale explained the largest proportion of variance in the models of life satisfaction and negative affect. In the model of positive affect, no differences were found regarding the proportion of variance explained by each of the SGRAT-NL subscale scores. (Table 7). Pearson’s correlation coefficients regarding the relationship between both gratitude questionnaires were *r* = 0.72 (*p* < .001) at T0 and *r* = 0.73 (*p* < .001) at T1.

**Table 8:** Pattern Matrix with loadings of SGRAT-NL items (N = 706).

Item	Factor 1	Factor 2	Factor 3

Item 15	0.797		
Item 6	0.773		
Item 10	0.735		
Item 3	0.701		
Item 11	0.678		
Item 2	0.512		
Item 8		0.790	
Item 1		0.722	
Item 14		0.711	
Item 5		0.659	
Item 13			0.723
Item 9			0.710
Item 4			0.613
Item 16			0.593
Item 7			0.557
Item 12			0.506

*Note.* Extraction Method: Maximum Likelihood. Rotation Method: Oblimin with Kaiser Normalization.

## Discussion

In this study, we examined the Dutch GQ6 and SGRAT regarding their factorial structure, the internal consistency and test-retest reliability of the (sub)scales, and the association of the (sub)scales with measures of well-being in a Dutch speaking adult sample. Parallel analyses, exploratory factor analyses, and confirmatory factor analyses found and confirmed the one-factor structure of the GQ6-NL as well as the three-factor structure of the SGRAT-NL. Internal consistency and test-retest reliability of both questionnaires and their subscales were good. In addition, our results showed that individuals with a stronger grateful disposition reported higher life satisfaction, higher positive affect, and less negative affect. The results showed that the total scores of the GQ6-NL and SGRAT-NL were significantly and positively associated with both life satisfaction and positive affect, indicating good convergent validity for both questionnaires. With regard to divergent validity, scores on both questionnaires were negatively associated with negative affect. We found a strong correlation between both scales indicating that the scales measure the same construct. However, the correlations were not perfect, possibly due to different conceptualizations of gratitude underpinning both scales.

Associations between the three subscales of the SGRAT-NL and measures of well-being were not assessed previously to the best of our knowledge. In our research, the subscale lack of a sense of deprivation showed a positive association with life satisfaction, a positive association with positive affect, and a negative association with negative affect, when controlled for the separate contribution of all other SGRAT-NL subscale measures. The association between lack of a sense of deprivation and life satisfaction corresponds with previous research on relative deprivation. Relative deprivation has been described as ‘the judgment that one is worse off compared to some standard and is accompanied by feelings of anger or resentment’ (Smith, Pettigrew, Pippin, & Bialosiewicz, 2012). This judgment may lead individuals to believe that they do not get what they deserve (Smith et al., 2012), and can result in increased negative affect, decreased positive affect and a decrease in feeling gratitude in life. The positive association of the subscale simple appreciation with life satisfaction and positive affect, and its negative association with negative affect supports these claims by suggesting that appreciation of the little things in life may increase positive feelings and life satisfaction, and reduces negative feelings. Interpretation of causality regarding these relationships is, however, hampered by the research design of the current study. The subscale appreciation of others showed no significant positive association with life satisfaction, a positive association with positive affect, and a positive association with negative affect. Wood, Maltby, Gillett, Linley, and Joseph (2008) stated that dispositional gratitude may lead to more conscious awareness about perceived social support. Because of this conscious awareness, it can be expected that appreciation of others would be positively associated to life satisfaction (a more evaluative state), than to positive and negative affect (emotional states). This positive association between perceived social support and life satisfaction has been found in previous research (Siedlecki, Salthouse, Oishi, & Jeswani, 2014). However, we found that appreciation of others is not related to life satisfaction, but seems to be associated with the experience of positive and negative emotions. The positive association with negative affect supports previous research that has shown gratitude to be not only related to positive affect, but also to negative affective experiences such as guilt and shame (McCullough, Emmons, Kilpatrick, & Larson, 2001), and indebtedness (Algoe, Gable, & Maisel, 2010; Watkins, Scheer, Ovnicek, & Kolts, 2006). Overall, our findings support that social components of gratitude are associated with both positive and negative affective experience.

There are some limitations of the current study that should be noted. First, the participants in this study were not randomly selected which may have led to a selective sample of adults. Furthermore, although the sample was demographically heterogeneous, participants who completed both measurements were higher educated, older, and showed less negative affect than those who dropped out after the baseline measurement. Although this may have introduced bias in the data, test-retest reliability was very good. Another limitation is that there is no direct comparison between the original and translated questionnaires within the same sample. However, to ensure an optimal translation of both questionnaires, the original versions were translated by bilingual translators to assure equivalence of meaning between both the translated and original versions.

Comparison of the outcomes of the SGRAT-NL with the outcomes of the GQ6-NL regarding reliability and validity in this study shows that there is great resemblance between both scales. The outcomes indicate that both scales are of sufficient psychometric quality to be used for assessment of the grateful disposition in individuals and groups (Kruyen, Emons, & Sijtsma, 2012). The choice between one scale or the other is therefore based on the amount of items, and on the different conceptualizations of both scales. The SGRAT-NL is based on three characteristics of individuals: lack of a sense of deprivation, simple appreciation, and appreciation of others; the GQ6-NL is based on four descriptive facets: intensity, frequency, span, and density.

As this is the first research using the subscales of the SGRAT-NL, future research is needed. Especially the subscales simple appreciation and appreciation of others should be scrutinized further. Simple appreciation seems to be associated with more positive affect and life satisfaction, and less negative affect; the results regarding appreciation of others were partly inconsistent with findings from previous research.

## Conclusion

The outcomes of our study replicated and extended previous studies (Froh et al., 2011; McCullough et al., 2002; Thomas & Watkins, 2003; Watkins et al., 2003), showing that the GQ6-NL and SGRAT-NL can be used to assess the grateful disposition in a Dutch speaking sample. The subscales of the SGRAT-NL showed good internal consistency and test-retest reliability and may be used for future research in order to further disentangle the relationship between a lack of a sense of deprivation, simple appreciation and the appreciation of others in the context of the grateful disposition.
